# DNAzymeBuilder, a web application for *in situ* generation of RNA/DNA-cleaving deoxyribozymes

**DOI:** 10.1093/nar/gkac269

**Published:** 2022-04-21

**Authors:** Razieh Mohammadi-Arani, Fatemeh Javadi-Zarnaghi, Pietro Boccaletto, Janusz M Bujnicki, Almudena Ponce-Salvatierra

**Affiliations:** Department of Cell and Molecular Biology & Microbiology, Faculty of Biological Science and Technology, University of Isfahan, Azadi Square, Hezar Jerib Avenue, 8174673441, Isfahan, Iran; Department of Cell and Molecular Biology & Microbiology, Faculty of Biological Science and Technology, University of Isfahan, Azadi Square, Hezar Jerib Avenue, 8174673441, Isfahan, Iran; Laboratory of Bioinformatics and Protein Engineering, International Institute of Molecular and Cell Biology in Warsaw, ul. Ks. Trojdena 4, PL-02-109 Warsaw, Poland; Laboratory of Bioinformatics and Protein Engineering, International Institute of Molecular and Cell Biology in Warsaw, ul. Ks. Trojdena 4, PL-02-109 Warsaw, Poland; Laboratory of Bioinformatics and Protein Engineering, International Institute of Molecular and Cell Biology in Warsaw, ul. Ks. Trojdena 4, PL-02-109 Warsaw, Poland

## Abstract

Nucleic acid cleaving DNAzymes are versatile and robust catalysts that outcompete ribozymes and protein enzymes in terms of chemical stability, affordability and ease to synthesize. In spite of their attractiveness, the choice of which DNAzyme should be used to cleave a given substrate is far from obvious, and requires expert knowledge as well as in-depth literature scrutiny. DNAzymeBuilder enables fast and automatic assembly of DNAzymes for the first time, superseding the manual design of DNAzymes. DNAzymeBuilder relies on an internal database with information on RNA and DNA cleaving DNAzymes, including the reaction conditions under which they best operate, their kinetic parameters, the type of cleavage reaction that is catalyzed, the specific sequence that is recognized by the DNAzyme, the cleavage site within this sequence, and special design features that might be necessary for optimal activity of the DNAzyme. Based on this information and the input sequence provided by the user, DNAzymeBuilder provides a list of DNAzymes to carry out the cleavage reaction and detailed information for each of them, including the expected yield, reaction products and optimal reaction conditions. DNAzymeBuilder is a resource to help researchers introduce DNAzymes in their day-to-day research, and is publicly available at https://iimcb.genesilico.pl/DNAzymeBuilder.

## Graphical Abstract

**Graphical Abstract:**
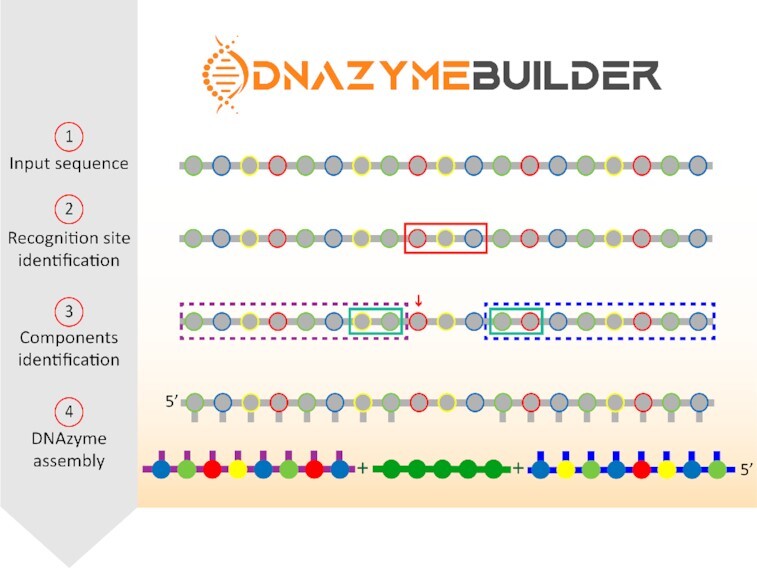
DNAzymeBuilder assembles nucleic acid cleaving DNAzymes for site-specific cleavage of RNA, DNA or chimeric substrates and provides detailed information on reaction conditions and products.

## INTRODUCTION

Deoxyribozymes, DNA catalysts or, simply, DNAzymes are single-stranded DNA molecules with catalytic properties. Inspired by the discovery of ribozymes in living organisms (1,2), and the subsequent isolation of artificial ribozymes in the laboratory (3,4), DNAzymes were introduced as synthetic catalysts in 1994 by Breaker and Joyce (5). Unlike their RNA counterparts, DNAzymes are not known to exist naturally, instead, they are identified through *in vitro* selection (6). Since the report of the first DNAzyme (5), DNAzymes catalyzing a growing variety of chemical reactions have been reported (7–19). Among them, RNA-cleaving DNAzymes have been vastly pursued due to their important applications in molecular (20) and synthetic biology (21,22), as therapeutic and diagnostic tools (23–26), as biosensors (27–29) and as molecular logic gates (30,31).

The field of DNAzymes has taken off thanks to DNAzymes’ desirable properties, for instance, inexpensive chemical synthesis (32), the possibility to isolate and optimize them via *in vitro* selection and *in vitro* evolution (33), trivial conjugation to nanomaterials (34), and increased chemical stability compared to protein enzymes and ribozymes (35). Recently, the structural characterization of three DNAzymes has proven that these catalysts adopt tertiary structures as complex as those of ribozymes and that are amenable to structure-guided engineering, which further increases their potential for day-to-day applications (36–38).

RNA- and DNA-cleaving DNAzymes can be tailor-made to the desired substrate thanks to their modular architecture. These DNAzymes comprise a catalytic core that is defined by its sequence or structure (39,40), flanked by two binding arms, which can be exchanged to specifically recognize the substrate. Nonetheless, in order to make use of them, it is necessary to define the appropriate binding arms and contrive the DNAzyme-substrate complexes manually. On top of that, it is fundamental to make the right DNAzyme choice, which entails finding the most appropriate one for cleaving the target sequence under the desired conditions and yielding the desired cleavage products. In summary, although DNAzymes have proven to be powerful and versatile tools, it remains difficult to make an informed choice when applying them to experiments given all the considerations and extensive literature review required from the experimentalists’ side. DNAzymeBuilder is a web application that makes RNA/DNA-cleaving DNAzyme assembly accessible and fast, taking over the time-consuming tasks necessary for DNAzymes manual design. DNAzymeBuilder relies on its back-end database and on the sequence provided by the user to automatically design DNA- and RNA-cleaving DNAzymes, providing a detailed output for each designed DNAzyme. DNAzymeBuilder is free and publicly available at the url: https://iimcb.genesilico.pl/DNAzymeBuilder.

## METHODS

### Data collection

The DNAzymeBuilder database contains information on 44 RNA- and 93 DNA-cleaving DNAzymes that are able to cleave in trans, i.e. cleavage occurs on a substrate that is independent from the DNAzyme, that is, in an intermolecular format. The collection includes RNA- and DNA-cleaving DNAzymes for which experimental data were published, and it will be updated as new data become available. DNAzymes that were not biochemically investigated or were reported to catalyze only self-cleavage (cleavage in *cis*), were not included. Self-cleaving DNAzymes usually contain a stem loop whose length and composition are rarely investigated. Therefore, there was not enough data to support the knowledge-based design of *cis*-cleaving DNAzymes. The catalytic cores included in the database comprise those of original DNAzymes, as well as catalytic core mutants (CM) that have resulted from *in vitro* evolution or by rational engineering of the former ones.

The information gathered in the back-end database of DNAzymeBuilder (4770 entries as of July 2021, accounting for each DNAzyme-substrate combination) includes recognition sites, contexts, cleavage sites, catalytic cores, binding arms, archetype of the binding arms, optimal reaction conditions, reaction *k*_obs_, reaction yield and reaction type (Supplementary Table S1). Such parameters may vary for a given DNAzyme, depending on the context in which the recognition site (RS) is positioned (Supplementary Table S2). These data were extracted from original research publications that report the *in vitro* selection of DNAzymes, or that describe their biochemical characterization in terms of substrate scope and optimal reaction conditions (Supplementary Table S3). A depiction of the variables used for the design of DNA- and RNA-cleaving DNAzymes can be found in Supplementary Figures S1 and S2, respectively.

### Algorithm specification

The DNAzymeBuilder algorithm searches for k-mers (a short specific set of nucleotides) that match a specific recognition site in its database (Supplementary Figure S3). While many DNAzymes only catalyze the cleavage reaction at a specific site within a particular sequence (10,41), others are capable of accepting a variety of recognition sequences (42). At the same time, a given substrate may contain hundreds of recognition sites for a variety of DNAzymes. DNAzymeBuilder searches for all possible recognition sites in the substrate that has been provided by the user and, once a RS is identified, the position of the recognition site start (RSS) is extracted for the calculation of the 5′ border of the right substrate-DNAzyme duplex and 3′ border of the left substrate-DNAzyme duplex. In addition, the sequence context is identified so that DNAzymeBuilder provides an estimate of the yield and *k*_ob__s_ of the reaction (Supplementary Figure S4), and decides on the binding arms archetype (Supplementary Figure S5).

### Implementation

The DNAzymeBuilder web application has been developed with Python 3.9 (https://www.python.org) scripting language, Django 3.2.4 framework (https://www.djangoproject.com/) and Datatables (https://datatables.net/) javascript library. The website, available at the web address https://iimcb.genesilico.pl/DNAzymeBuilder presents a simple graphical user interface browsable utilizing the menu bar present in the upper part of the pages.

## RESULTS AND DISCUSSION

DNA- and RNA-cleaving DNAzymes are powerful and versatile catalysts that have useful applications in therapeutics, nanotechnology, environmental monitoring, and molecular biology. They offer practical advantages with respect to ribozymes and protein enzymes, e.g. they can operate over a wide range of pH and temperature, but most importantly, they can be tailored to cleave any nucleic acid substrate at specific sites.

Our group has recently published DNAmoreDB, a database that collects general information on all DNAzymes reported to date (43). More than 1500 sequences have been gathered in DNAmoreDB among which RNA-cleaving DNAzymes are the most represented ones (1028 sequences). Recent examples of their applications include a logic gate for the detection of SARS-CoV-2, based on the combined actions of the 8–17 DNAzyme and exonuclease III (31). Also, xeno-nucleic-acid versions of the well-known 10–23 DNAzyme have been applied in a SARS-CoV-2 detection system (44) and, for efficient gene-silencing *in vivo* (21).

In spite of their wide potential, it is often very difficult to select the best possible DNAzyme for a particular substrate because its biochemical characterization may have been published in multiple papers over the years rather than in a single publication. Moreover, a particular DNAzyme may not be efficiently performing the cleavage reaction for the substrate of choice or under the conditions intended for the experiment.

DNAzymeBuilder enables the automatic choice and assembly of nucleic acid cleaving DNAzymes for the first time, representing a programmatic solution to the manual design of DNAzymes. DNAzymeBuilder relies on an internal database that contains information on all the relevant parameters necessary to choose and assemble the best cleaving DNAzymes given the substrate and preferences specified by the user.

DNAzymeBuilder homepage introduces the website and its functionalities. The DNAzymeBuilder page hosts the tool that allows users to find DNAzymes that cleave the input substrate based on its sequence. DNAzymeBuilder accepts DNA, RNA and chimeric substrate sequences with a maximum length of 2000 nucleotides in FASTA, EMBL, GenBank, or RAW formats. IUPAC codes for specified nucleotides (A, C, G, T and U) and incompletely specified nucleotides (R, Y, M, K, S, W, H, B, V, D and N) are accepted in the input sequence; however, only specified nucleotides are considered for DNAzyme assembly. In other words, DNAzymeBuilder will not consider cleavage sites, recognition sites or contexts containing nucleotides, such as Y, H or D. Non-IUPAC letters are deleted from the substrate, since no complementary nucleotides are defined for them. The user must specify the type of substrate, otherwise DNA is set as the default. It is also possible to transform the input sequence into its reverse complement, and to convert the DNA sequence into RNA, and vice versa. Users can narrow down the options to choose from by specifying additional parameters such as the cleavage position on the substrate, the length of the binding arms, their melting temperature, the reaction mechanism, the cofactor employed by the DNAzyme, the desired pH range, or even the DNAzyme that should carry out the reaction; otherwise, default parameters are used.

**Figure 1.**
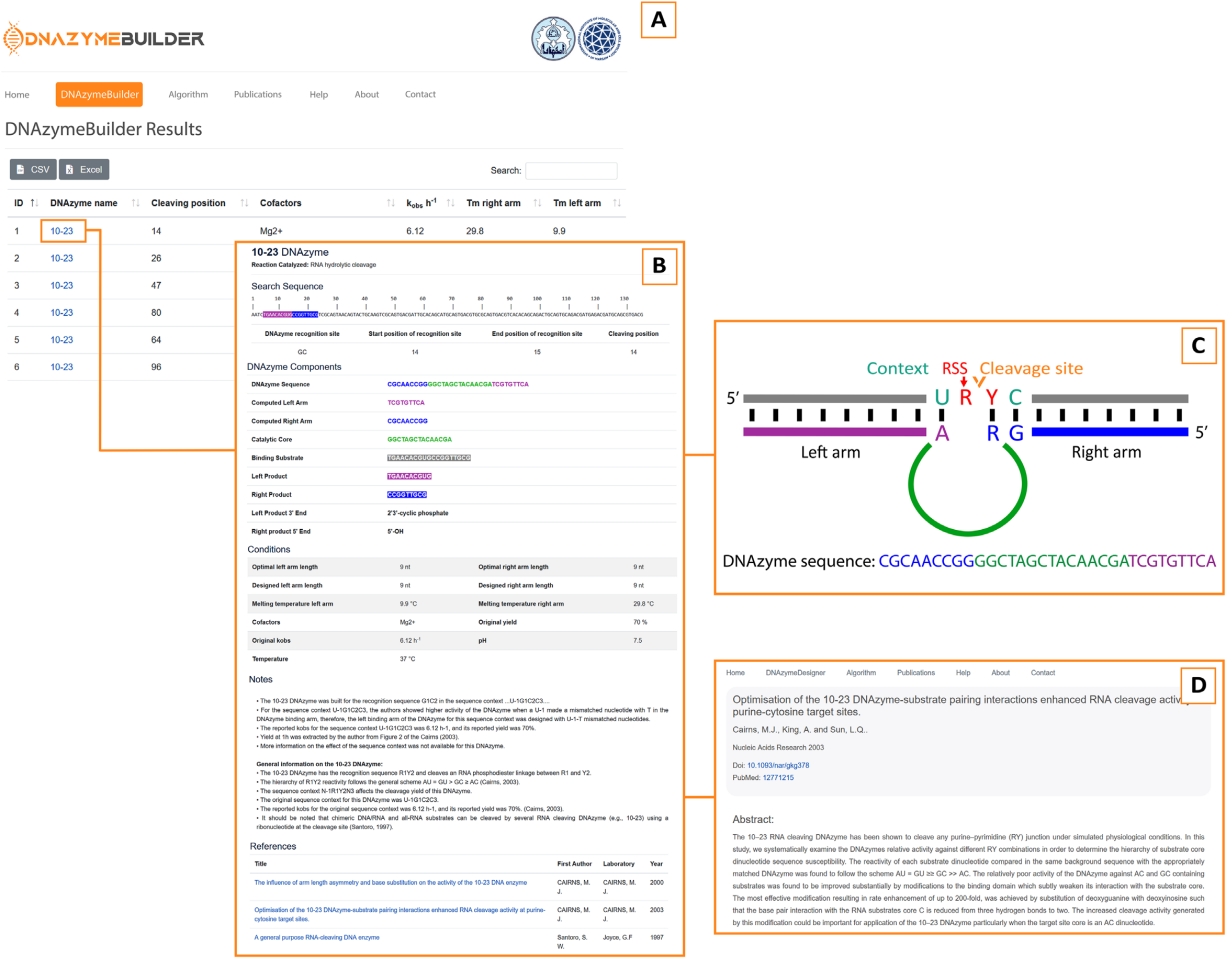
DNAzymeBuilder's *Results* page. The assembled DNAzymes are shown with a summary of relevant parameters, such as cleavage position, *k_obs_*, binding arms’ Tm and cofactors (inset **A**). Detailed information for each assembled DNAzyme is accessible after clicking on it (inset **B**). The detailed results page includes a color-coded schematic view of the DNAzyme (inset **C**) and references to the original research articles related to the DNAzyme (inset **D**).

DNAzymeBuilder outputs a list of candidate DNAzymes to carry out the cleavage reaction according to the parameters set by the user (Figure 1). The DNAzymes appear listed in a table, with their name, cleavage site position, cofactors, *k*_obs_ and binding arms’ Tm. These results may be downloaded in CSV or Excel format.

Clicking on each of the resulting DNAzymes reveals detailed information for the catalyst. The target sequence appears highlighted in the regions that are complementary to the binding arms, and the RS and RSS are indicated. Below, a table with the full-length assembled sequence, its components, and reaction products is displayed. A color-coded schematic of the DNAzyme-substrate complex serves as visual help to understand how these components relate to one another. Finally, a table with the reaction conditions and kinetic parameters is presented. Since the chosen DNAzymes may cleave RS within sequence contexts that differ from those originally reported, DNAzymeBuilder reports estimated relative yields and rates of these ‘mutant forms’ with respect to the original ones utilizing its database. These estimations appear as RRk (Relative Reported *k*_obs_), RRY (Relative Reported Yield) and RCY (Relative Calculated Yield) in the notes section (Supplementary Table S1 and Supplementary Figure S4).

The Algorithm tab explains how DNAzymeBuilder works (Supplementary Figure S3), including two example videos featuring the steps required for DNAzymes choice and assembly, the relevant sequence elements, and the different criteria according to which DNAzymeBuilder generates its output. The Publications page contains all the references from which data were extracted to build the DNAzymeBuilder database. The Help page contains a video tutorial on how to use DNAzymeBuilder, a glossary of terms used throughout the website, descriptors of the parameters reported by DNAzymeBuilder in the DNAzymes result pages, and a schematic depiction of possible arrangements for DNAzymes binding arms. Finally, the About page provides information on how to find us, while the Contact page allows users to directly contact us with issues related to DNAzymeBuilder.

DNAzymeBuilder is an online resource that automatically finds and assembles RNA- and DNA-cleaving DNAzymes with the scope of providing users with the best possible choice of DNAzyme based on their preferences. Manual design of DNAzymes is time-consuming and prone to human error; therefore, a web application that supersedes such an approach, can help researchers introduce DNAzymes in their day-to-day research to further unleash DNAzymes potential and applications. Future versions of DNAzymeBuilder will be updated with new DNAzymes as data becomes available, so that it is as much of a comprehensive resource as possible. Besides, our group is currently working on an extension of DNAzymeBuilder that will tackle DNAzymes that catalyze additional reaction types.

## DATA AVAILABILITY

The web interface to the service is available at https://iimcb.genesilico.pl/DNAzymeBuilder. This website is free, open to all users and no login or password is required.

## Supplementary Material

gkac269_Supplemental_File
