# Supplementary material for: DNAzymeBuilder, a web application for in situ generation of RNA/DNA-cleaving deoxyribozymes
Source: Nucleic Acids Res. 2022 Apr 21;50(W1):W261–5. doi: 10.1093/nar/gkac269 (PMC9252740; doi:10.1093/nar/gkac269)
Supplement: gkac269_Supplemental_File [file gkac269_supplemental_file.pdf]

Supplementary data

DNAzymeBuilder, a web application for *in situ* generation of RNA/DNA-cleaving deoxyribozymes

Razieh Mohammadi-Arani[1], Fatemeh Javadi-Zarnaghi[1,*], Pietro Boccaletto[2], Janusz M. Bujnicki[2,*] and Almudena Ponce-Salvatierra[2,*]

[1] Department of Cell and Molecular Biology & Microbiology, Faculty of Biological Science and Technology, University of Isfahan, Azadi Square, Hezar Jerib Avenue, 8174673441, Isfahan, Iran

[2] Laboratory of Bioinformatics and Protein Engineering, International Institute of Molecular and Cell Biology in Warsaw, ul. Ks. Trojdena 4, PL-02-109 Warsaw, Poland

* To whom correspondence should be addressed. Tel: (+48-22) 597-07-50; Fax: (+48-22) 597-07-15; Email: aponce-salvatierra@iimcb.gov.pl.

Correspondence may also be addressed to Fatemeh Javadi-Zarnaghi (fa.javadi@sci.ui.ac.ir) and to Janusz M. Bujnicki (janusz@iimcb.gov.pl).

Table of contents:

Table S1. Description of the parameters that have been collected in the DNAzymeBuilder database.

Table S2. A part of the DNAzymeBuilder database used for the assembly of an exemplary DNAzyme with various contexts.

Table S3. References from which the content of the database has been retrieved.

Figure S1. Application of the database variables for the assembly of three DNA-cleaving DNAzymes: F-8, A-2, and PLDz.

Figure S2. Application of the database variables for the assembly of three RNA-cleaving DNAzymes: 8-17, 10-23, and VMA8.

Figure S3. Work diagram of DNAzymeBuilder.

Figure S4. DNAzymeBuilder reports on expected kinetic parameters for an exemplary DNAzyme.

Figure S5. Interaction between DNAzymes and their substrates

Table S1. Description of the parameters that have been collected in the DNAzymeBuilder database.

Variable	Definition
Recognition site (RS)	A recognition site designates the required sequence on the substrate that is recognized by the DNAzyme. It mostly encompasses the cleavage site, although this is not the case for some DNAzymes. Most DNAzymes have only one RS but some have more.
Recognition site start (RSS)	The starting point of the recognition site. The first nucleotide in the recognition sequence is marked as RSS and it serves as the starting point to calculate the borders/length/boundaries of the DNAzyme's binding arms.
Cleavage site (CS)	The cleavage site represents the exact position of the phosphodiester bond that is cleaved. For DNAzymes that only cleave once, the phosphodiester bond is defined by specifying the flanking nucleotides. For DNAzymes that cleave more than once, the excised nucleotides are marked as the cleavage site.
Context	The nucleotides upstream and downstream from the RS constitute the context, which influences the activity of the DNAzyme.
Left and right binding arms	The binding arms of the DNAzyme are the sequences to the left and right of the catalytic core acting as scaffolds for the substrate. They are assembled based on the RSS point and their optimal lengths are chosen based on the data reported in the original articles. Nonetheless, users may specify the binding arms' length, or select it based on the desired Tm.
Archetype of the binding arms	The binding arms of the DNAzyme may be i) fully complementary to the substrate, ii) contain mismatches, or iii) bulges. A binding arm may also acquire a triplex structure in combination with the substrate.
Catalytic core	The catalytic core sequence is specific to each DNAzyme. However, some DNAzymes may still be catalytically active in mutant forms. If such mutants are known to catalyze the reaction as efficiently as the parent DNAzyme, the output of DNAzymeBuilder includes both the originally reported and the mutant catalytic cores (CM).
Optimal reaction conditions	The reaction conditions include the buffer components, cofactors, and the reaction temperature. Reaction conditions influence the kinetics of DNAzymes. DNAzymeBuilder reports the conditions under which the DNAzyme's activity is optimal. If such information was not available, the reaction conditions reported by DNAzymeBuilder are those under which the *in vitro* selection was carried out.
Experimental reaction yield	The yield of the cleavage reaction is calculated with the formula Yield = band intensity of the product/(band intensities of the substrate+product). This equation employs experimental data i.e. band intensities that have been visualized and quantified from denaturing polyacrylamide gels to calculate yield at specific time points.
reaction k_{obs} and Y_{max}	Ymax is the maximum possible product reached in a reaction at infinite time. Reaction k_{obs} is the observed kinetic rate of the reaction. Both values are obtained by fitting the formula Yield= $Y_{max}*(1–e^{-kobs*t})$ to the original cleavage data (experimental reaction yield).

Relative Reported k_{obs}	RRk = reported k_{obs} mutant form/k_{obs} original
Relative Reported Yield	RRY (specific time) = reported Y mutant form /Y original
Relative Calculated Yield	RCY (specific time) = calculated Y mutant form / Y original

Table S2. A part of the DNAzymeBuilder database used for the assembly of an exemplary DNAzyme with various contexts. The recognition site for F-8 deoxyribozymes is known to consist of a TGC sequence. One nucleotide prior and after this sequence were shown to affect the catalytic efficiency of the DNAzyme as well as the binding arms' architecture. When a thymidine is present at the 5' of the recognition site, the best catalytic activity was reported for DNAzymes that accommodate a T.G wobble pair just at the 3' end of the left binding arm, thereby, the DNAzymes were assembled to be mismatched at the left binding arm for contexts that start with thymidine. The presented yields in the table were extracted from M. Wang et al. (1), Figures S7.1, S7.2 and, S7.3.

types	optimal right arm length	optimal left arm length	start right arm 1	end right arm 1	start left arm 1	end left arm 1	start left arm 2	end left arm 2	left unpaired nucleotide	context	start right product	end right product	start left product	end left product	cofactor	original k_{obs}	left cleavage products	right cleavage products	original yield	context yield
match	9	12	2	11	-12	0	0	0		CTGCG	1	11	-12	0	Mn^{2+} Cu^{2+}	0.14	3'-phosphate	5'-phosphate	81	81
match	9	12	2	11	-12	0	0	0		GTGCG	1	11	-12	0	Mn^{2+} Cu^{2+}	0.14	3'-phosphate	5'-phosphate	63	6
match	9	12	2	11	-12	0	0	0		GTGCA	1	11	-12	0	Mn^{2+} Cu^{2+}	0.14	3'-phosphate	5'-phosphate	81	NR
match	9	12	2	11	-12	0	0	0		GTGCC	1	11	-12	0	Mn^{2+} Cu^{2+}	0.14	3'-phosphate	5'-phosphate	81	NR
match	9	12	2	11	-12	0	0	0		GTGCT	1	11	-12	0	Mn^{2+} Cu^{2+}	0.14	3'-phosphate	5'-phosphate	81	NR
match	9	12	2	11	-12	0	0	0		ATGCG	1	11	-12	0	Mn^{2+} Cu^{2+}	0.14	3'-phosphate	5'-phosphate	79	20
match	9	12	2	11	-12	0	0	0		ATGCA	1	11	-12	0	Mn^{2+} Cu^{2+}	0.14	3'-phosphate	5'-phosphate	81	NR
match	9	12	2	11	-12	0	0	0		ATGCC	1	11	-12	0	Mn^{2+} Cu^{2+}	0.14	3'-phosphate	5'-phosphate	81	NR
match	9	12	2	11	-12	0	0	0		ATGCT	1	11	-12	0	Mn^{2+} Cu^{2+}	0.14	3'-phosphate	5'-phosphate	81	NR
match	9	12	2	11	-12	0	0	0		CTGCC	1	11	-12	0	Mn^{2+} Cu^{2+}	0.14	3'-phosphate	5'-phosphate	81	36
match	9	12	2	11	-12	0	0	0		CTGCA	1	11	-12	0	Mn^{2+} Cu^{2+}	0.14	3'-phosphate	5'-phosphate	81	72
match	9	12	2	11	-12	0	0	0		CTGCT	1	11	-12	0	Mn^{2+} Cu^{2+}	0.14	3'-phosphate	5'-phosphate	81	NR
mismatch	9	12	2	11	0	0	-12	-1	G	TTGCG	1	11	-12	0	Mn^{2+} Cu^{2+}	0.14	3'-phosphate	5'-phosphate	80	52
mismatch	9	12	2	11	0	0	-12	-1	G	TTGCA	1	11	-12	0	Mn^{2+} Cu^{2+}	0.14	3'-phosphate	5'-phosphate	80	NR
mismatch	9	12	2	11	0	0	-12	-1	G	TTGCC	1	11	-12	0	Mn2+ Cu2+	0.14	3'-phosphate	5'-phosphate	80	NR
mismatch	9	12	2	11	0	0	-12	-1	G	TTGCT	1	11	-12	0	Mn2+ Cu2+	0.14	3'-phosphate	5'-phosphate	80	NR

Table S3. References from which the content of the database has been retrieved.

DNAzyme	Publications from which the data was extracted	DNAzyme	Publications from which the data was extracted
**F-8**	10.1093/nar/gku592	**7ZJ7**	10.1021/co300111f
**F-8 (x)**	10.1093/nar/gku592, 10.1016/j.biochi.2019.07.022	**7ZJ12**	10.1021/co300111f
**F-8 (ATP)**	10.1093/nar/gku592	**9ZL1**	10.1021/co300111f
**F-8(CM1)**	10.1093/nar/gku592	**8VA2**	10.1021/co300111f
**F-8(CM2)**	10.1093/nar/gku592	**8VA4**	10.1021/co300111f
**F-8(CM3)**	10.1093/nar/gku592	**8VA8**	10.1021/co300111f
**F-8(CM4)**	10.1093/nar/gku592	**8VA10**	10.1021/co300111f
**F-8(CM5)**	10.1093/nar/gku592	**8VA11**	10.1021/co300111f
**F-8(CM6)**	10.1093/nar/gku592	**8VA23**	10.1021/co300111f
**6YR25**	10.1093/nar/gkr860	**8VA25**	10.1021/co300111f
**7VH6**	10.1093/nar/gkr860	**8VB1**	10.1021/co300111f
**7VK55**	10.1093/nar/gkr860	**8VB2**	10.1021/co300111f
**8VL51**	10.1093/nar/gkr860	**8VB5**	10.1021/co300111f
**9NL27**	10.1021/bi1013672, 10.1039/c0cc04575f	**8VB7**	10.1021/co300111f
**9NL1**	10.1021/bi1013672	**8VB9**	10.1021/co300111f
**9NL12**	10.1021/bi1013672	**8VB12**	10.1021/co300111f
**9NL33**	10.1021/bi1013672	**8VB16**	10.1021/co300111f
**8NLJ1**	10.1021/bi1013672	**8VB18**	10.1021/co300111f
**10MD-AC**	10.1039/c0cc04575f	**8VB22**	10.1021/co300111f
**10MD5**	10.1038/nchembio.201, 10.1021/bi1013672	**8VB25**	10.1021/co300111f
**A-2**	10.1039/c6ob00148c	**8VB26**	10.1021/co300111f
**A-3**	10.1039/c6ob00148c	**7YE2**	10.1021/co300111f
**A-3(CM1)**	10.1039/c6ob00148c	**7YE5**	10.1021/co300111f
**A-3(CM2)**	10.1039/c6ob00148c	**7YE8**	10.1021/co300111f
**A-3(CM3)**	10.1039/c6ob00148c	**7YE19**	10.1021/co300111f
**A-3(CM4)**	10.1039/c6ob00148c	**6YF13**	10.1021/co300111f

**A-3(CM5)**	10.1039/c6ob00148c	**8YM4**	10.1021/co300111f
**I-R3**	10.1021/ja403585e, 10.1021/acscatal.8b01466	**6YF2**	10.1021/co300111f
**II-R1**	10.1021/ja403585e	**6YF15**	10.1021/co300111f
**RadDz3**	10.1021/jacs.6b10274	**6YF16**	10.1021/co300111f
**RadDz3(CM1)**	10.1021/jacs.6b10274	**6YF20**	10.1021/co300111f
**RadDz3(CM2)**	10.1021/jacs.6b10274	**6YF27**	10.1021/co300111f
**RadDz6**	10.1021/jacs.6b10274	**8YG7**	10.1021/co300111f
**6YJ10**	10.1039/C2SC01067D	**8YG1**	10.1021/co300111f
**6YJ14**	10.1039/C2SC01067D	**8YG24**	10.1021/co300111f
**6YL4**	10.1039/C2SC01067D	**8YG29**	10.1021/co300111f
**6YL11**	10.1039/C2SC01067D	**8YM17**	10.1021/co300111f
**6YL24**	10.1039/C2SC01067D	**8YM20**	10.1021/co300111f
**6YL34**	10.1039/C2SC01067D	**8YM26**	10.1021/co300111f
**7YK24**	10.1039/C2SC01067D	**8-17**	10.1073/pnas.94.9.4262, 10.1093/nar/gkm1175
**7YK34**	10.1039/C2SC01067D	**10-23**	10.1089/oli.1.2000.10.323, 10.1093/nar/gkg378, 10.1073/pnas.94.9.4262
**7YK35**	10.1039/C2SC01067D	**Ce13d**	10.1021/ac403762s, 10.1007/s00239-015-9715-7
**11PC5**	10.1093/nar/gkr860	**NaA43**	10.1073/pnas.1420361112
**13PD1**	10.1093/nar/gkr860	**17E**	10.1093/nar/28.2.481, 10.1021/acs.analchem.5b00220
**13PB2**	10.1093/nar/gkr860	**6SE3**	10.1021/ja4032488
**10-12opt**	10.1038/s41598-019-44750-x	**6SE10**	10.1021/ja4032488
**8VA5**	10.1021/co300111f	**6SE11**	10.1021/ja4032488
**8VA6**	10.1021/co300111f	**6SE12**	10.1021/ja4032488
**8VB4**	10.1021/co300111f	**6SE15**	10.1021/ja4032488
**8VB20**	10.1021/co300111f	**6SE20**	10.1021/ja4032488
**8VB21**	10.1021/co300111f	**6SE22**	10.1021/ja4032488
**7ZG2**	10.1021/co300111f	**6SE30**	10.1021/ja4032488
**7ZG5**	10.1021/co300111f	**9SE20**	10.1021/ja4032488
**9ZH5**	10.1021/co300111f	**9SE22**	10.1021/ja4032488
**9SH25**	10.1021/ja4032488	**9SE23**	10.1021/ja4032488
**9SK1**	10.1021/ja4032488	**9SE33**	10.1021/ja4032488

**9SK2**	10.1021/ja4032488	**9SH2**	10.1021/ja4032488
**9SK5**	10.1021/ja4032488	**9SH3**	10.1021/ja4032488
**9SK17**	10.1021/ja4032488	**9SH5**	10.1021/ja4032488
**9SK29**	10.1021/ja4032488	**9SH18**	10.1021/ja4032488
**9SK31**	10.1021/ja4032488	**VMA8**	10.1002/anie.201808745
**Dz15WS_E**	10.1002/open.201600141	**AA07**	10.1002/anie.202006218
**Dz27WS_E**	10.1002/open.201600141	**AA14**	10.1002/anie.202006218
**HD2**	10.1073/pnas.95.11.6027	**AA17**	10.1002/anie.202006218
**Ag10c**	10.1021/acs.analchem.6b00327	**AC17**	10.1002/anie.202006218
**G5_mini**	10.1016/1074-5521(94)90014-0	**NaH1**	10.1002/cbic.201800322
**VMC10**	10.1002/anie.201808745	**Tm7**	10.1093/nar/gku1296
**VMA15**	10.1002/anie.201808745	**Lu12**	10.1021/ac5029962
**PLDz**	10.1073/pnas.95.5.2233, 10.1016/s0968-0896(01)00035-9, 10.1016/j.isci.2020.101555, 10.1016/j.bios.2015.03.070		

F-8

Recognition sequence: $T_1G_2C_3$

Context: $C_{-1}T_1G_2C_3G_4$

Left arm: [-12 : 0]

Right arm: [2 : 11]

Left product: [-12 : 0]

Right product: [1 : 11]

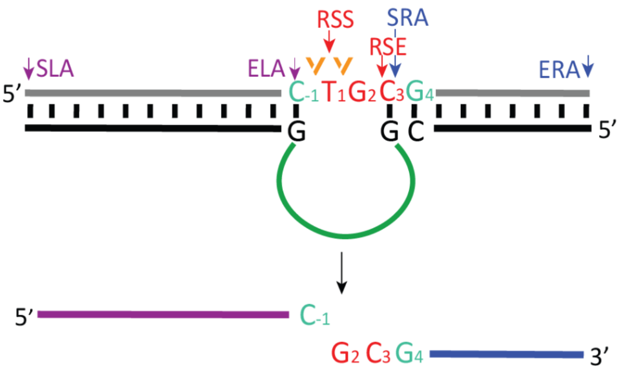

A-2

Recognition sequence: $A_1C_2T_3G_4$

Context: $T_{-1}A_1C_2T_3G_4C_5G_6T_7$

Left arm: [-6 : -1]

Right arm_1: [5 : 6]

Mismatched nucleotide right arm: C

Right arm_2: [7 : 11]

Left product: [-6 : 6]

Right product: [7 : 11]

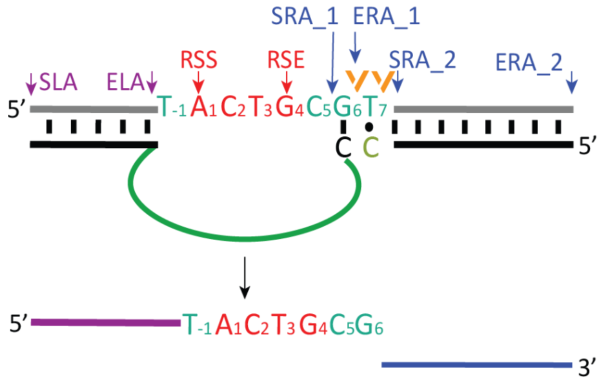

PLDz

Recognition sequence: $A_1A_2T_3A_4C_5G_6$

Context: $T_{-4}T_{-3}C_{-2}T_{-1}$

Left arm: CA + [0 : -4] + TTT + complement [-4 : 0]

Right arm: [5 : 15]

Left product: [-4 : 6]

Right product: [7 : 15]

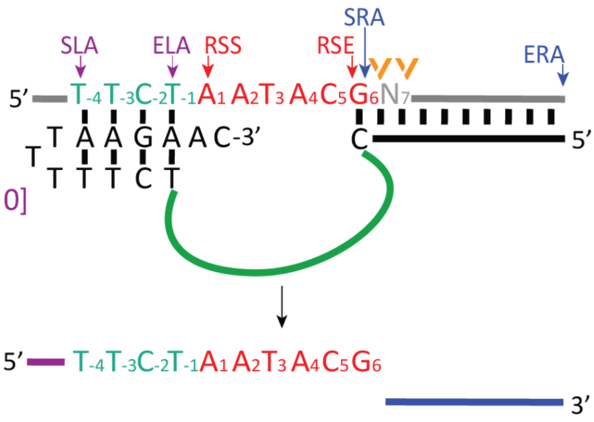

Figure S1. Application of the database variables for the assembly of three DNA-cleaving DNAzymes: F-8, A-2, and PLDz. SLA: start left arm, ELA: end left arm, RSS: recognition site start, RSE: recognition site end. SRA: start right arm, ERA: end right arm, orange arrows: cleavage site(s).

8-17

Recognition sequence: G_1G_2
Context: -
Left arm: [-10 : 0]
Right arm_1: [0 : 0]
Unpaired nucleotide right arm: T
Right arm_2: [2 : 11]
Left product: [-10 : 1]
Right product: [1 : 11]

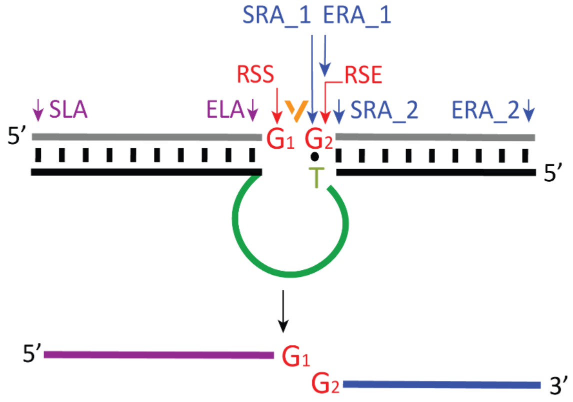

10-23

Recognition sequence: G_1U_2
Context: $C_{-1}G_1U_2U_3$
Left arm: [-9 : 0]
Right arm: [1 : 10]
Left product: [-9 : 1]
Right product: [1 : 10]

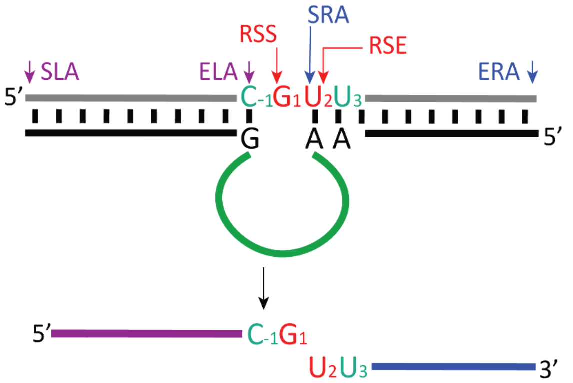

VMA8

Recognition sequence: $G_1G_2A_3C_4U_5$
Context: $A_{-1}G_1G_2A_3C_4U_5U_6$
Left arm: [-9 : 1]
Right arm_1: [3 : 4]
Mismatched nucleotide right arm: G
Right arm_2: [5 : 13]
Left product: [-9 : 1]
Right product: [1 : 13]

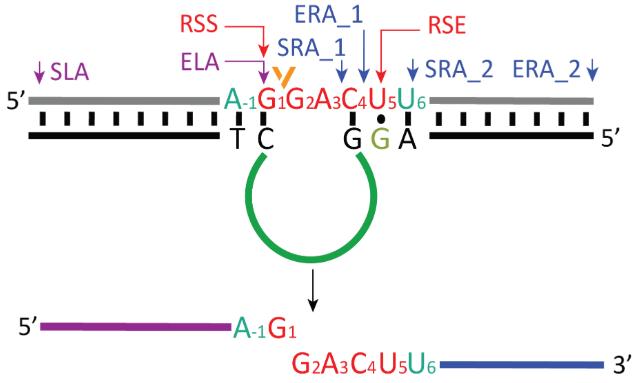

Figure S2. Application of the database variables for the assembly of three RNA-cleaving DNAzymes: 8-17, 10-23, and VMA8. SLA: start left arm, ELA: end left arm, RSS: recognition site start, RSE: recognition site end. SRA: start right arm, ERA: end right arm, orange arrows: cleavage site(s).

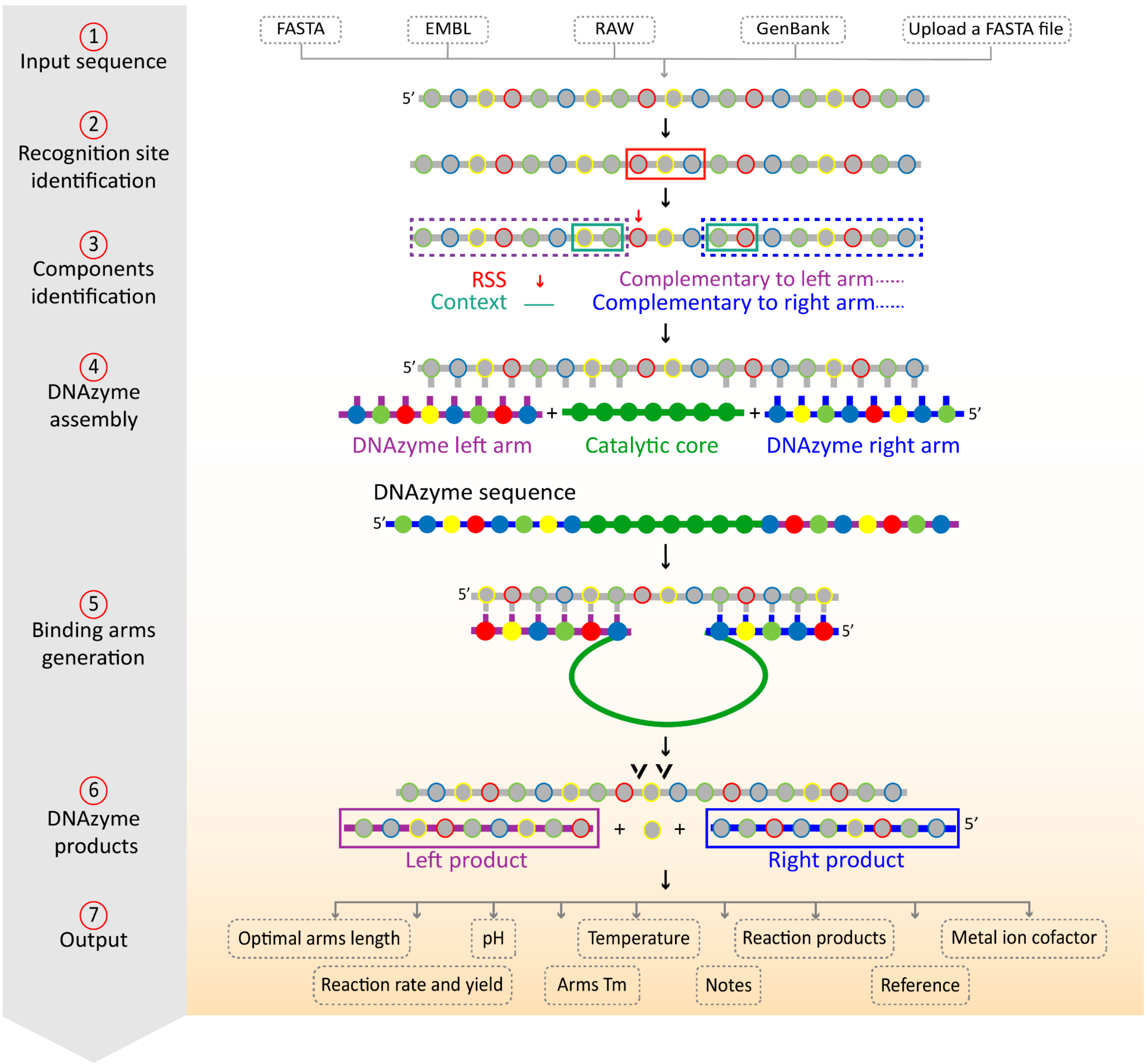

Figure S3. Work diagram of DNAzymeBuilder. The user can provide the target RNA/DNA sequence in FASTA, EMBL, GenBank, and RAW formats in the specified box, or upload the sequence file in FASTA format. Once the recognition site is identified, DNAzymeBuilder defines the remaining components necessary for DNAzymes assembly. The right and left arms of the DNAzyme are designed to hybridize to the substrate, and the catalytic core is then placed between the two arms. The length of the binding arms may be defined in three different ways: by default (as reported in literature/optimal length), according to a user-specified length, and according to a user-specified Tm. The resulting DNAzyme sequence is written in 5' to 3' direction. Comprehensive data for each generated DNAzyme, including optimal reaction conditions, becomes available on the Results page. This figure exemplifies the assembly of DNA catalysts that catalyze two adjacent cleavage reactions with the excision of a nucleotide. The case of DNAzymes catalyzing one cleavage reaction is not illustrated in this figure, however, it would be analogous to the example above, excluding the excised nucleotide.

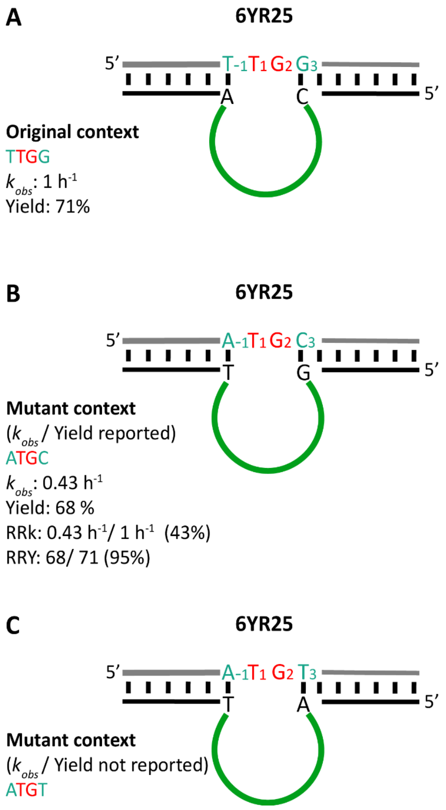

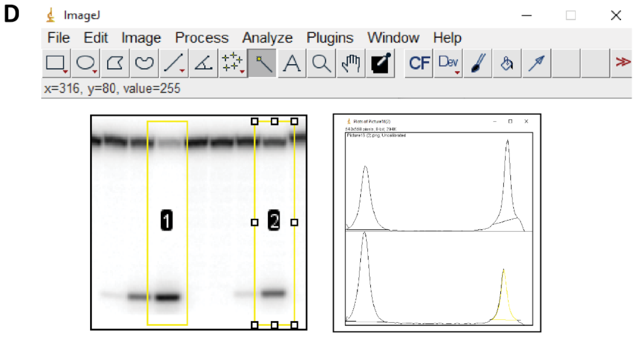

E

$$\text{Yield} = \frac{\text{Intensity of the product's bond}}{\text{Intensity of the product's bond} + \text{Intensity of the substrate's bond}}$$

$$\text{RCY} = \frac{\text{Yield mutant form}}{\text{Yield original}}$$

F

Contexts	Bands	Band intensities	Yield	RCY	Score
Original	Substrate band<br>Product band	6825.953<br>5365.64	0.4401	100	*****
Mutant	Substrate band<br>Product band	9325.368<br>3540.619	0.2752	62	****

Figure S4. DNAzymeBuilder reports on expected kinetic parameters for an exemplary DNAzyme. The kinetic parameters gathered in DNAzymeBuilder for each DNAzyme-substrate pair (ca. 4770 entries) match one of the situations depicted in A, B, or C. **A)** The substrate contains an exact match to the originally reported RS and context: the reported k_{obs} and yield are those that were originally reported in the literature for that DNAzyme. **B)** The substrate contains a RS and/or context that varies at some position(s) with respect to the originally reported one, but there is kinetic data available for such mutant(s) available in the literature: the reported k_{obs} and yield are those that were reported in the literature. In addition, we report the relative reported k_{obs} (RRk) and relative reported yield (RRY) values, which relate directly to the values reported in situation A. For instance, the original substrate of 6YR25 bears T-1 and G3 surrounding the T1G2 recognition site. The T>A mutation at position -1, and G>C mutation at position 3 affect the yield and k_{obs} of the reaction according to Xiao. et. al. (2). The available literature data for such mutants were used to calculate RRk and RRY for the specific context of A-1T1G2C3. **C)** No kinetic data was reported for that specific RS and/or context but gel images were available in the literature: For example, the specific context A-1T1G2T3. In such cases, we report the relative calculated yield (RCY), as described in panels D, E, and F. **D)** In order to calculate RCY, ImageJ (3) was utilized to estimate band intensities from gel images. **E)** Band intensities were applied to the yield formula and RCY was calculated as the ratio of the mutant yield to the original yield. **F)** an example of how RCY was calculated.

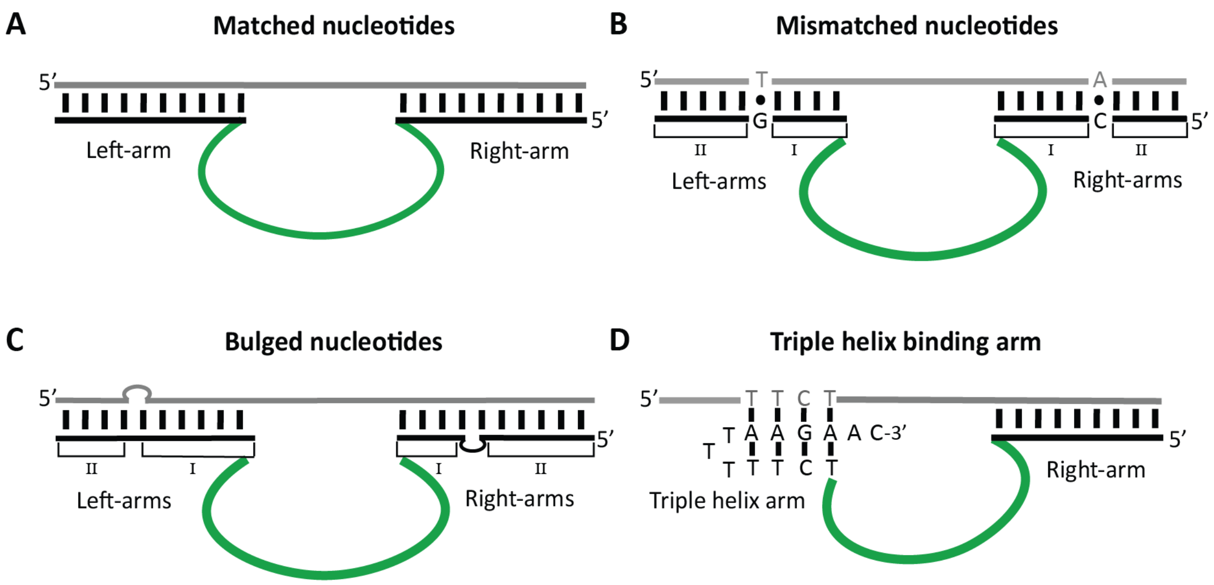

Figure S5. Interaction between DNAzymes and their substrates. According to the literature, most DNAzymes have duplex DNA binding arms, and the presence of matched (A), mismatched (B), or bulged nucleotides (C) in the arms of some DNAzymes enhances their catalytic activity. The PLDz DNAzyme displays a left arm with a characteristic triple helix structure (D).

References

1. Wang,M., Zhang,H., Zhang,W., Zhao,Y., Yasmeen,A., Zhou,L., Yu,X. and Tang,Z. (2014) In vitro selection of DNA-cleaving deoxyribozyme with site-specific thymidine excision activity. *Nucleic Acids Res.*, **42**, 9262–9269.
http://www.ncbi.nlm.nih.gov/pmc/articles/PMC4132718
https://doi.org/10.1093/nar/gku592

2. Xiao,Y., Wehrmann,R.J., Ibrahim,N.A. and Silverman,S.K. (2012) Establishing broad generality of DNA catalysts for site-specific hydrolysis of single-stranded DNA. *Nucleic Acids Res.*, **40**, 1778–1786.
http://www.ncbi.nlm.nih.gov/pmc/articles/PMC3287185
https://doi.org/10.1093/nar/gkr860

3. Schneider,C.A., Rasband,W.S. and Eliceiri,K.W. (2012) NIH Image to ImageJ: 25 years of image analysis. *Nat. Methods*, **9**, 671–675.
http://www.ncbi.nlm.nih.gov/pmc/articles/PMC5554542
https://doi.org/10.1038/nmeth.2089
